# Improvement of Polytetrafluoroethylene Membrane High-Efficiency Particulate Air Filter Performance with Melt-Blown Media

**DOI:** 10.3390/polym13234067

**Published:** 2021-11-23

**Authors:** Euijin Shim, Jeong-Phil Jang, Jai-Joung Moon, Yeonsang Kim

**Affiliations:** 1 Advanced Textile R&D Department, Korea Institute of Industrial Technology (KITECH), 143 Hanggaulro, Sangnok-gu, Ansan-si 15588, Korea; pelice35@kitech.re.kr; 2 Department of Advanced Organic Materials Engineering, Jeonbuk National University, 567 Baekje-daero, Deokjin-gu, Jeonju-si 54896, Korea; jpjang@cands.kr; 3 Clean and Science Co., Ltd., 67, 3sandan 3-gil, Buk-myeon, Jeongeup-si 56137, Korea; jjmoon@cands.kr

**Keywords:** HEPA filter, melt-blown, pre-filter, PTFE membrane, clean room

## Abstract

Polytetrafluoroethylene (PTFE) membrane filters are widely used in low-load application areas, such as industrial cleanrooms, due to their low initial pressure drop. In this study, melt-blown (MB) nonwoven was introduced as a pre-filtration layer at the front end of a high-efficiency particulate air (HEPA) filter to improve the filter performance of the PTFE membrane. Pre-filtration reduces the average particle size, which reaches the PTFE membrane and reduces the dust load on the HEPA filters. A comparative analysis of the HEPA filters by composite MB and PTFE was conducted. Regarding the MB composite on the PTFE, low-weight and high-weight MB composites were effective in increasing dust filtration efficiency, and the dust loading capacity of the PTFE composite with high-weight MB increased by approximately three times that of the PTFE membrane. In addition, the filter was installed on an external air conditioner in an actual use environment and showed a high efficiency of 99.984% without a change in differential pressure after 120 days.

## 1. Introduction

High-efficiency particulate air (HEPA) filters have been used in various fields to maintain a clean air environment for industrial facilities, such as producing semiconductor and LCD panels, bio-pharmacies, and nuclear power plants [1,2,3]. In general, glass fibre has been applied to HEPA filter media. However, polytetrafluoroethylene (PTFE) membranes used to remove particles in industries have recently been developed to have the same efficiency level as glass fibre. PTFE has emerged as an alternative option with a much lower initial pressure drop [3,4].

PTFE membranes exhibit excellent chemical resistance, thermal stability, and strong hydrophobicity owing to the strong C–C and C–F bonds. The combination of strong bonds, a protective sheath, and non-polarity not only makes PTFE thermally stable but also inactive. These features are suitable for a variety of applications, such as fine dust filtration, exhaust gas treatment, and membrane distillation. PTFE, a microporous membrane, is a valuable air filter medium with a high filtration efficiency and flow velocity [5,6]. The porous membrane filter material refers to many small pores in the membrane matrix, such as the expanded PTFE (ePTFE) membrane. Owing to its low porosity, this type of material has low airflow and generally has an exceedingly high filtration effect but extremely high air resistance [7,8].

The HEPA filter determines the filtration performance by the HEPA filter media, which is characterised by two parameters: filtration efficiency and pressure drop. These parameters increase as dust accumulates in the filter medium. Long-term pressure drop performance is important, especially for filter performance, as increased pressure drop affects increased energy consumption and determines filter life [3]. Thus, the dust loading capacity is a particularly important parameter in filter design. It is necessary to consider the particle loading characteristics at the filter design stage [9,10].

Previous researchers developed a three-layer filter in which each layer showed a different fibre density to achieve high collection efficiency and low pressure drop and reported the filtration performance dust holding capacity of the filter [10,11].

Although there are studies on layering melt-blown nonwoven (hereafter MBNW) as a pre-filter [12,13], no study has applied MB as a pre-filter to PTFE membranes to demonstrate their high filter characteristics. Thus, this study compared the pre-filter effect of the HEPA filter produced by filtering the PTFE membrane under MB production conditions. The pre-filter reduces the average particle size that reaches the PTFE membrane while reducing the dust load on the HEPA filter [14,15]. The basic properties, such as thickness, weight, and air permeability, of the filter media were analysed. This study quantified the consequential effects of the overall pressure drop and filter lifespan. Finally, the industrial application was confirmed through demonstration experiments in a real usage environment.

## 2. Materials and Methods

### 2.1. Materials

MBNW media samples were produced from commercial grade polypropylene (PP). PP resin (melt index: 1200 g/10 min) was purchased from LG Chem Co., Ltd. (Seoul, Korea). A melt blowing pilot line from Clean and Science Co was used, and the PTFE membrane was produced by Micro-One Co. (Cheonan, Korea) The average thickness of the PTFE membrane was 4.33 μm. Low-melting polyethylene terephthalate (PET) and a nonwoven PE/PET blend were used as the PTFE support.

### 2.2. Preparation of the High-Efficiency Particulate Air Filter with Melt-Blown Nonwoven

**Figure 1:**
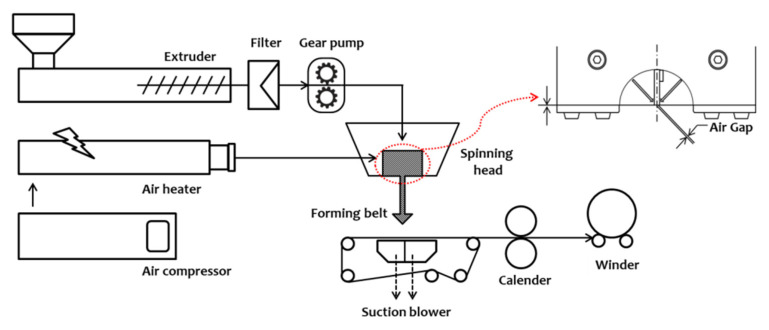
Schematic of a melt-blown spinning process.

MBNW is a major process used to manufacture fibrous nonwoven fabrics. It was used as a pre-filter to prevent nanoparticles from passing through the PTFE membrane layer. The PP-based MBNW fabric was prepared using a pilot-scale melt-injection setup (Figure 1). The process temperature of the extruder was controlled using a two-component nozzle to 200–250 °C. The MB-grade PP pellets were placed into a twin-screw extruder, and the output was controlled by the speed of the gear pump. The melted polymer was sprayed in the form of single fibres and solidified from the collector. In addition, the net was electrically charged on the MB side by a corona discharger to further improve the filtration efficiency. Four types of MBNW media, as detailed in Table 1, were tested in this study. Finally, the HEPA filter media were fabricated with the MB pre-filter layer and PTFE membrane layer.

The manufactured MBNW and PTFE membranes were laminated to form composites. The HEPA filter unit to be installed in a commercial heating, ventilation, and air conditioning (HVAC) system was fabricated from a PTFE/MB composite pleated and assembled into an aluminium frame.

### 2.3. Characterization

The physical properties of the PTFE membrane and MB composite, such as thickness, pore size, and air permeability, were evaluated. Their thickness was determined using a thickness tester (No.20465, Mitutoyo Co., Kawasaki, Japan) according to the ASTM D 5729-9 standard. The pore sizes distribution of these specimens were measured using a capillary flow porometer (CFP-1500-AEX, PMI Inc., Ithaca, NY, USA) according to the ASTM F316-03 standard. Air permeability was measured using an air permeability tester (FX3300, TexTest Instruments, Zürich, Switzerland) at a constant pressure drop of 125 Pa. Surface images of the PTFE membrane and MB were observed by field emission scanning electron microscopy (FE-SEM; SU8010, Hitachi Co., Tokyo, Japan) with an acceleration voltage of 10 kV after sputter coating with osmium (Os). The static water contact angle (WCA) of the filter media was determined following the sessile drop method using a contact angle analyzer (DSA100, Krüss Co., Hamburg, Germany).

### 2.4. Filtration Test

The efficiencies of the HEPA filter were evaluated using different types of particles and measuring instruments.

First, the filtration efficiency and resistance of the PTFE and PTFE/MB composite filter media were measured using an automated particulate filtration tester (TSI Inc., Shoreview, MN, USA). Poly(α-olefin) (PAO) was selected as the experimental particle and represented oily aerosol dust. The tests were based on the EN 1822 Part 3 standards. The filtration efficiency (*E*) was calculated based on the aerosol concentration upstream (*C_up_*) and the aerosol concentration downstream (*C_down_*) as follows [16,17]:

*E* = (*C_up_* − *C_down_*)/*C_up_* (1)

Second, for PTFE membrane and PTFE/MB composite media, the dust loading pattern at 2 wt.% concentration of NaCl aerosol and 5.33 (cm/s) face velocity was determined to investigate the effect of the MB pre-filtration layer and MB weight on the HEPA filter loading process. An experiment on the dust load performance comparison experiment between PTFE/MB composite media and PTFE only media was conducted, where NaCl solid particles were loaded for 60 min under the same operating conditions. 

Third, HVAC environment tests were conducted in an external air conditioner (1680 cmm) environment. The wind speed was 56 cmm, and after the initial differential pressure and efficiency were measured, their changes were observed at regular intervals. The tests were measured according to the EN 1822 Part 1 standards. The efficiency was calculated based on the Equation (1) for 0.3 μm particles. In this study, the results were measured and presented after 120 days [18].

## 3. Results and Discussion

### 3.1. Effect of Melt-Blown Physical Properties for High-Efficiency Particulate Air Filter 

In this study, the physical properties according to weight were compared and analysed before MBNW was introduced into the PTFE filter media. This is due to the fact that the MB structure, porosity, and air permeability are factors that can affect filter performance, that is, differential pressure and efficiency [13,19].

#### 3.1.1. Morphology Structure

**Figure 2:**
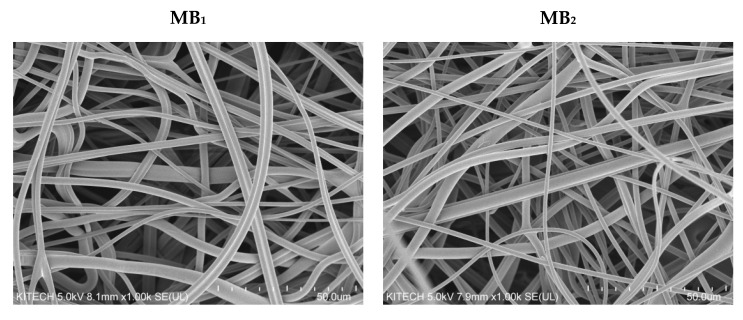

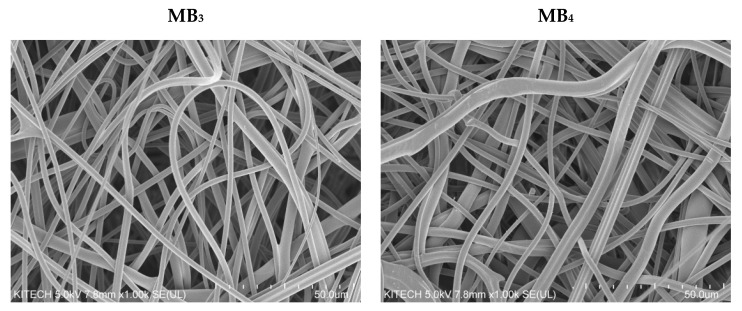
Specifications and SEM images (×1000) of melt-blown media by various weight.

SEM micrographs were obtained at a magnification of 1000 (Figure 2). The bonds of the fibres can be observed in the SEM images. It can also be observed that the diameters of the fibres formed in the process are diverse, as observed in the typical MB webs of other polymers. The fibre orientation was random depending on the processing conditions used. Fibre tangles are successfully obtained at high levels, which affect the pore size required for filtration applications and other controlled barrier fabrics [19]. In addition, it was confirmed that the higher the weight, the higher the density observed with the naked eye.

#### 3.1.2. Contact Angle

The MBNW pre-filter material was analysed using a contact angle measuring instrument. The measurement results of the contact angle of MBNWs are shown in Table 2. The contact angle is the angle formed when the surface comes into contact with the liquid forming MBNW-liquid interface. Based on these measurements, surfaces are classified into hydrophilic, hydrophobic and super-hydrophobic types. Super-hydrophobicity is a phenomenon in which the contact angle of the surface exceeds 150°. Hydrophobicity is a phenomenon in which the contact angle of the surface exceeds 90° [20,21]. The average contact angle of the four MBNW types used in this study was 132.4~146.6°. All four MBNWs made of polypropylene showed hydrophobic properties. As the weight of the MB increased, the contact angle also increased.

#### 3.1.3. Pore Size

The MBNW pre-filter materials were analysed using a pore-size analyser. The corresponding curve between flow and pressure was a ‘wet curve’, and a dry test was conducted to obtain a ‘dry curve’. The two curves and semi-dry curves show the pore characteristics of the sample, and the cumulative distribution curve of the pore size can be calculated by accumulating the flow [22,23]. The average pore size and pore size distribution of MBs are shown in Table 3. The average pore sizes were between 10.25 and 12.54 μm, and the bubble pore sizes ranged from 20.03–24.56 μm. The heavier the weight of the MB, the smaller the mean pore size and bubble pore size. The weight and pore size were inversely proportional. 

The pore sizes of the PTFE and MB composites were compared and analysed (Table 4). Only PTFE without MB was measured with an average pore size of 1.08 μm and a bubble pore size of 8.13 μm. It was confirmed that the average pore size when combined with MB was smaller than that when PTFE alone was used inside and outside. Although the pore size of MB alone was larger than that of PTFE, it was confirmed that adding a layer reduces the pore size of the entire filter media. This is the expected result of MB acting as a pre-filtration layer.

#### 3.1.4. Air Permeability

**Figure 3:**
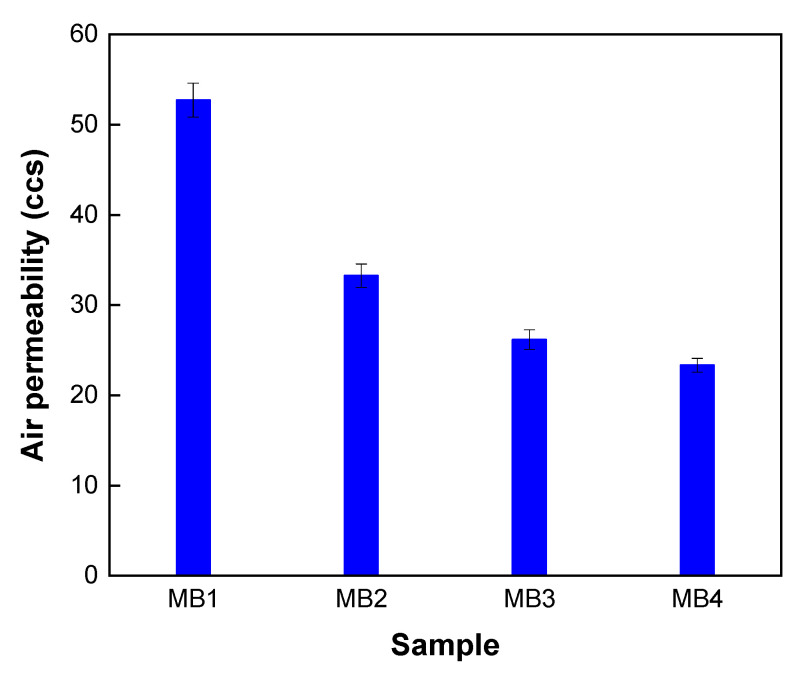
Air permeability of MB by various weights.

**Figure 4:**
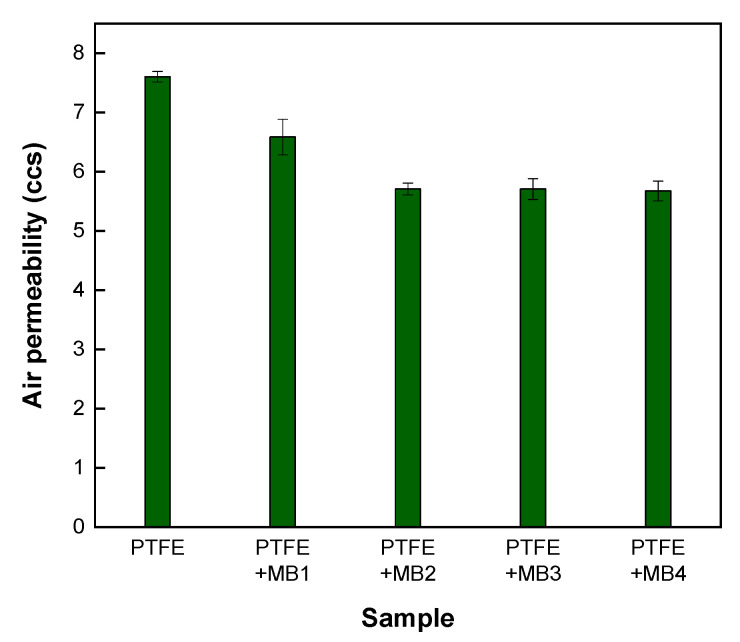
Air permeability of PTFE only and PTFE with MB composites.

The air permeability of nonwoven fabrics is an important property that depends on the openness or porosity of the structure. This greatly adjusts the thermal comfort of fabrics. Melt-blown webs have much smaller fibre diameters than spun bond fabrics, which results in smaller pore sizes and lower pore volumes and lower air permeability. However, the air permeability values are still sufficiently high for most applications [13,24]. Generally, higher porosity leads to increased permeability. In this study, as the weight increased, the air permeability decreased, and the pore size influenced the air permeability, even when compared with the pore size results. Multiple parameters, such as fibre microstructure, fibre diameter, and nonwoven pore size, affect the air permeability of elastomeric nonwoven composites [13]. Thus, before applying MB to PTFE filter media, the structural characteristics and air permeability of MB were analysed. As shown in Figure 3, the air permeability of MB was inversely proportional to its weight, and the difference in air permeability between MB_1_ and MB_4_ was approximately 30 ccs. Figure 4 shows the air permeability of MB when combined with a PTFE membrane. In the result with only MB, the air permeability was significantly reduced below 8 ccs, which is determined by the effect of the PTFE membrane, and was 7.6 ccs when only the membrane was measured. By composite with MB on the PTFE membrane, the air permeability was reduced compared to when only PTFE was used. However, this was determined introduce the MB layer, similar to the result of pore size. Additionally, when MB was combined with a PTFE membrane, there was no significant difference in weight compared to MB alone at 6.5 ccs for PTFE with MB_1_ composite and 5.6 ccs for PTFE with the MB_4_ composite.

### 3.2. Filtration Performance

**Figure 5:**
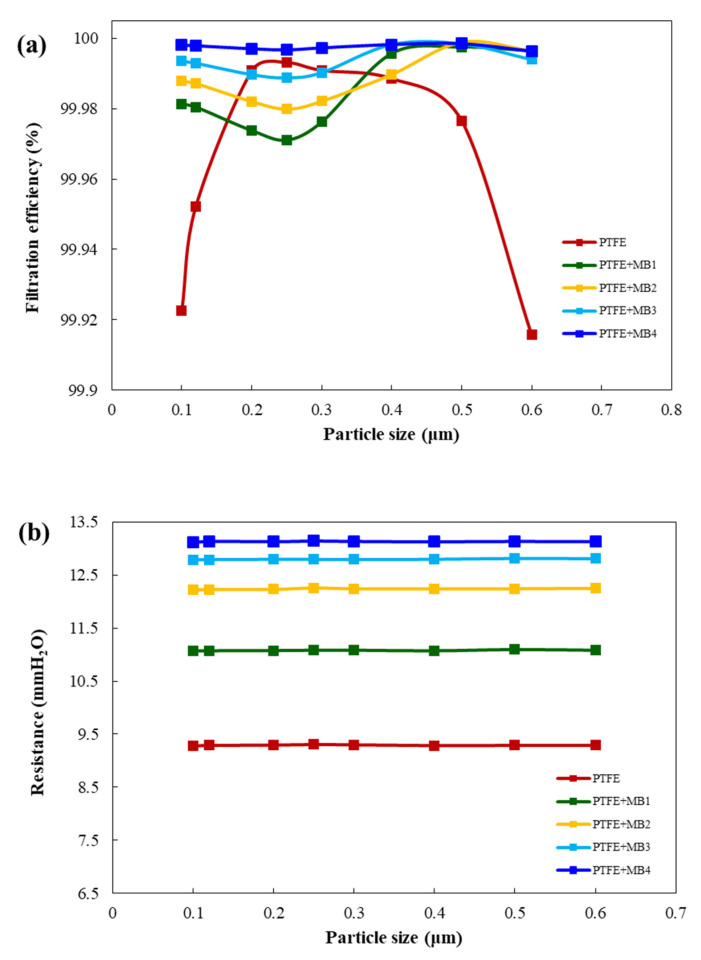
(**a**) Efficiency and (**b**) resistance of the PTFE membrane/MB composite on different test dust particle sizes to confirm the MB weight effects according to the EN1822 standards.

Figure 5 shows the performance evaluation measurements of the filtration efficiency by particle sizes from 0.1 to 0.6 μm. The filter performance was compared and analysed according to MB weight. The optimal MB weight should be verified since it is the quality factor for determining the pressure drop and filtration efficiency of the filter [25]. During the evaluation of filtration quality, the pressure drop is particularly important. Conventional PTFE HEPA filters provide high particle removal efficiency. However, filter design must consider such that the pressure drop can increase quickly as accumulated particles clog the filter pores [23,26,27].

The nanoparticle filtration efficiency and resistance of the PTFE membrane and MB composite increased as the MB weight increased. This confirmed that the higher the MB weight, the smaller the size of the particles. These results suggest that the degree of competition in the size of dust molecules also affects the filtration efficiency, and that the filtration efficiency can be increased owing to the electrostatic force of MB [25,28].

### 3.3. Dust Loading Test

Dust loading experiments were conducted at 5.3 cm/s face velocity and 2 wt.% aerosol concentrations. The dust loading test results are important parameters of filter load characteristics related to a higher dust retention capacity life, which means a longer life and both a lower energy consumption and cost of the filter media [15,29].

**Figure 6:**
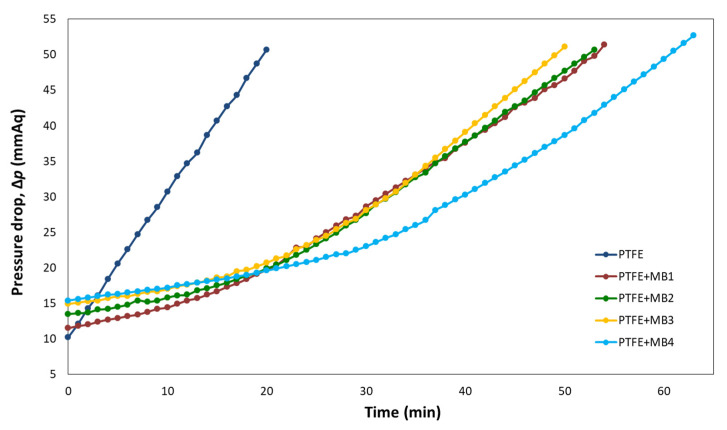
Dust loading performance of PTFE and MB composite at various MB weights.

Figure 6 shows the results of the dust loading performance, and Table 5 shows the amount collected after the loading test. As can be seen in Figure 6 and Table 5, the pressure drop increased modestly when pre-filtration was performed with the MB composite compared to the PTFE membrane only. In addition, when only PTFE was used, the dust collection per area was 1.0. However, when MB was introduced, it increased to a minimum of 3.0 and a maximum of 5.9. A higher dust loading capacity indicates that the prevailing pressure drop occurs more slowly [30,31]. Therefore, among MB_1_-MB_4_, MB_4_ had the highest dust retention capacity. With the introduction of MB, the filter is expected to last longer with high efficiency.

### 3.4. Filter Performance Evaluation in Heating, Ventilation, and Air Conditioning System

The performance was compared by installing a sample in which MB_4_ was compounded with PTFE membrane media, the existing glass media, and PTFE media in a commercial HVAC system. As presented in Table 6, the glass media had a slightly higher PTFE media efficiency but had the limitation of applying differential pressure compared to the increased efficiency. The PTFE media developed by improving the shortcomings of glass media, greatly reducing the differential pressure. However, it was not very efficient [3]. It was confirmed in the mounting test that the PTFE media sample in which MB was compounded compensated for these drawbacks and improved the efficiency to a low differential pressure. In addition, the differential pressure and efficiency were measured after 15, 60, and 120 days when the filter was installed to check the condition of the filter. After 120 days, the efficiency of the MB and PTFE composite samples showed efficiencies of 99.984%, which was higher than the glass media which showed an efficiency of 99.978%. Even the MB and PTFE composites showed good filter performance with a differential pressure about two times lower than that of the glass media. It is expected that the performance as a long-life, high-efficiency HEPA filter will be confirmed and that it will be used in an actual industrial environment.

## 4. Conclusions

In this study, MB was introduced into the pre-filter to improve the performance of the PTFE membrane based HEPA filter. Four types of MB were produced and analysed according to weight. The comparative analysis of MB_1_, MB_2_, MB_3,_ and MB_4_ showed that the filter efficiency was effectively increased when MB_4_ was introduced into the PTFE membrane. It was confirmed that the DHC performance of PTFE and MB composite samples was increased by approximately three times or more compared to the case where only the PTFE membrane was used, and the life of the filter could be extended. In addition, when evaluating the performance in the actual filter use environment, the differential pressure did not increase even after 120 days, and the efficiency was excellent at 99.984%. As the PTFE membrane-based HEPA filter with MBNW shows excellent performance, thus benefiting the excellent performance and long life of the filter in various HEPA filter usage environments.

## Figures and Tables

**Table 1:** Characteristics of tested melt-blown media.

Sample	Weight (gsm)	Thickness (μm)	Fibre Diameter (μm)
MB_1_	10	55 ± 3	1.80 ± 0.42
MB_2_	15	79 ± 2	1.61 ± 0.29
MB_3_	20	99 ± 6	1.92 ± 0.29
MB_4_	22	110 ± 2	1.97 ± 0.33

gsm: gram per square meter.

**Table 2:** Contact angle of melt-blown media by various weight.

	MB_1_	MB_2_	MB_3_	MB_4_
Contact angle (°)	132.4 ± 0.3	139.2 ± 0.5	142.1 ± 0.4	146.6 ± 0.2
Image	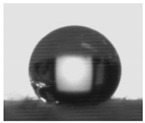	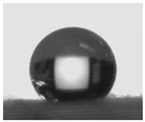	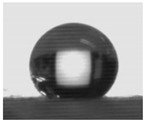	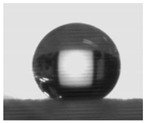

**Table 3:** Pore size of melt-blown media by various weight.

Analysis	MB_1_	MB_2_	MB_3_	MB_4_
Mean pore size diameter (μm)	12.54	11.22	10.25	10.47
Bubble pore size diameter (μm)	24.56	22.08	21.06	20.03

**Table 4:** Pore size of PTFE with melt-blown media by various weights.

Analysis	PTFE	PTFE + MB_1_	PTFE + MB_2_	PTFE + MB_3_	PTFE + MB_4_
Mean pore size diameter (μm)	1.08	0.85	0.81	0.85	0.74
Bubble pore size diameter (μm)	8.13	7.49	6.12	8.45	8.18

**Table 5:** Dust loading capacity.

	PTFE	PTFE + MB_1_	PTFE + MB_2_	PTFE + MB_3_	PTFE + MB_4_
Dust loading capacity (g/m^2^)	1.0	3.0	3.8	4.4	5.9

**Table 6:** Filter performance on contaminated air (outdoor fresh air conditioner: 1680 cmm, Air flow: 56 cmm).

	Glass Media		PTFE Media		PTFE + MB Composite	
	Pressure Drop (mmAq)	Efficiency (%)	Pressure Drop (mmAq)	Efficiency (%)	Pressure Drop (mmAq)	Efficiency (%)
Initial measurement	28.8	99.981	14.8	99.934	14.5	99.961
After 15 days	28.7	99.983	15.2	99.926	14.6	99.967
After 60 days	28.3	99.993	16.5	99.966	14.6	99.986
After 120 days	28.4	99.978	17.0	99.968	14.6	99.984

Efficiency for 0.3 μm particles.

## Data Availability

Data available in a publicly accessible repository.
